# Nitrogen-Doped Carbon Encapsulated Partial Zinc Stannate Nanocomposite for High-Performance Energy Storage Materials

**DOI:** 10.3389/fchem.2021.769186

**Published:** 2021-11-18

**Authors:** Jiage Yu, Zhijie Liu, Xian Zhang, Yu Ding, Zhengbing Fu, Feng Wang

**Affiliations:** School of Chemistry and Materials Science, Hubei Engineering University, Xiaogan, China

**Keywords:** partial zinc stannate, nitrogen-doped carbon, core–shell structure, high-performance energy storage materials, battery

## Abstract

As a bimetal oxide, partial zinc stannate (ZnSnO_3_) is one of the most promising next-generation lithium anode materials, which has the advantages of low operating voltage, large theoretical capacity (1,317 mA h g^−1^), and low cost. However, the shortcomings of large volume expansion and poor electrical conductivity hinder its practical application. The core-shell ZnSnO_3_@ nitrogen-doped carbon (ZSO@NC) nanocomposite was successfully obtained by coating ZnSnO_3_ with polypyrrole (PPy) through *in situ* polymerization under ice-bath conditions. Benefiting from this unique compact structure, the shell formed by PPy cannot only effectively alleviate the volume expansion effect of ZnSnO_3_ but also enhance the electrical conductivity, thus, greatly improving the lithium storage performance. ZSO@NC can deliver a reversible capacity of 967 mA h g^−1^ at 0.1 A g^−1^ after 300 cycles and 365 mA h g^−1^ at 2 A g^−1^ after 1,000 cycles. This work may provide a new avenue for the synthesis of bimetal oxide with a core–shell structure for high-performance energy storage materials.

## Introduction

The 14th Five-Year Plan points out that the key core technologies of new energy vehicles should be broken through to make pure electric vehicles become the mainstream. First of all, the development of power Li-ion battery is one of the key core technologies to be addressed (Zhang et al., 2020a; Peng et al., 2021; Yu et al., 2021). At present, the capacity of commercial graphite anode materials has been developed to the ceiling (the theoretical lithium storage capacity is 372 mA h g^−1^), and it is urgent to develop a new generation of power anode materials, such as ZnO, SnO, Co_3_O_4_, and so on (Wang et al., 2019; Zhou et al., 2019; Xie et al., 2020). For the special bimetal oxide structure, Zinc stannate (ZnSnO_3_) has more advantages than single metal oxides. For instance, ZnSnO_3_ possesses higher theoretical capacity (1,317 mA h g^−1^) and lower working potential than that of SnO_2_ (Zhou et al., 2017; Ma et al., 2018; Tan et al., 2018; Wen et al., 2019). However, the shortcomings of large volume expansion and poor electrical conductivity hinder its commercial application.

Recently, various reports (Liu et al., 2018; Wang et al., 2020a; Kong et al., 2021) revealed that it is an effective strategy to address these problems by constructing the core–shell structure, with the active material as the core and the conductive carbon layer as the shell. For example, Tao Liu et al. (2018) presented a facile and efficient template method for the synthesis of core–shell Co_3_O_4_/nitrogen-doped carbon hollow spheres, which deliver an excellent electrochemical performance. Kuaibing Wang et al. (2020a) reported a successful and effective strategy to prepare core-shell nickel/nitrogen codoped carbon (Ni@NC) materials. The as-prepared material serving as both the positive and negative electrodes, deliver excellent electrochemical performance. Fanjun Kong et al. (2021) obtained the core–shell SnSe@C with high performance by electrospinning, which display superior cycling and rate performance owing to this compact core–shell structure. All these examples show that the core-shell structure with carbon material as the shell can effectively reduce the volume expansion effect and greatly improve the electrochemical performance. Among them, the choice of shell material is crucial. For the low cost, stable voltage window, excellent mechanical resilience and high electronic conductivity (10–50 S cm^−1^), polypyrrole (PPy) is one of the star conductive coating layer materials (Ma et al., 2019; Wang et al., 2020b; Zhang et al., 2020b; Chu et al., 2020). Jun-Hong Zhang et al. (2020b) constructed a core–shell NiO-NiMoO_4_/PPy composite. The flexible PPy cannot only construct a pathway and promote electrons transferring on the active materials but also can serve as the mechanical protection film to refrain from the pulverization of the active materials. What is more, it confirmed that the PPy modification was a direct and effective strategy to improve the reversible capacity and cycling performance of active materials.

Herein, inspired by the above work and our previous study, the PPy modification strategy is adopted to prepare the ZSO@NC nanocomposite. The core–shell ZSO@NC nanocomposite was successfully obtained by coating ZnSnO_3_ with PPy through *in situ* polymerization under ice-bath conditions. The as-prepared ZnSnO_3_@NC nanocomposites display superior cycling and rate performances, owing to this unique compact core–shell structure.

## Experimental Section

### Synthesis of ZnSn(OH)_6_ Precursors and Pure Zinc Stannate

The 6-mmol SnCl_4_•5H_2_O and 24-mmol NaOH were dissolved in 60 ml of ultra-pure water and stirred in an ice bath for 60 min. Then, 30 ml of 0.2 mmol/L ZnSO_4_•7H_2_O solution was slowly added into the reaction container under continuous agitation. At this time, white suspended matter would gradually appear, indicating the formation of ZnSn(OH)_6_. After centrifugation and washing operation, the product was placed in a vacuum drying oven and dried for 10 h to get the ZnSn(OH)_6_ powder. The precursor powder was calcined at 500°C for 2 h at a heating rate of 5°C/min under a protective atmosphere. Finally, the pure ZnSnO_3_ powders were obtained.

### Synthesis of ZSO@NC

The 0.4 g of ZnSn(OH)_6_ was dispersed in 200 ml of deionized water by ultrasonic manipulation. Then, 0.5 ml of pyrrole monomer was quickly added to the beaker under the ice-bath environment, followed by 200 ml of 0.02 M ammonium persulfate solution slowly added, and continuous stirring for 12 h. The above samples were successively washed by water and ethanol three times, and then filtered and dried in a vacuum drying oven at 80°C. Finally, under inert gas protection, the samples were heated to 500°C at 2°C/min for 2 h in a tubular furnace to obtain NC@ZSO-2. As the control group, different volumes of pyrrole monomers were coated in this experiment. In the experiment, 0.25 ml of pyrrole was added to get NC@ZSO-1, and 1 ml of pyrrole was added to get NC@ZSO-3. The corresponding original contributions presented in the study are included in Supplementary Material.

### Synthesis of ZnO-SnO_2_@NC and Nitrogen-Doped Carbon

Except for the different calcination conditions, the preparation process of ZnO-SnO_2_@NC is identical with that of ZSO@NC-2. ZnO-SnO_2_@NC is obtained at 800°C for 2 h at a heating rate of 5°C/min under a protective atmosphere. Similarly, nitrogen-doped carbon (NC) is prepared by imitating the production process of ZSO@NC-2 without adding zinc source and tin source.

## Results and Discussion

**FIGURE 1:**
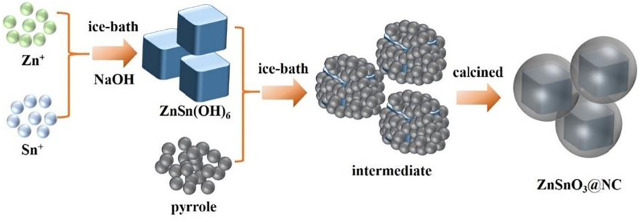
Schematic illustration of the synthesis process of ZSO@NC.

**FIGURE 2:**
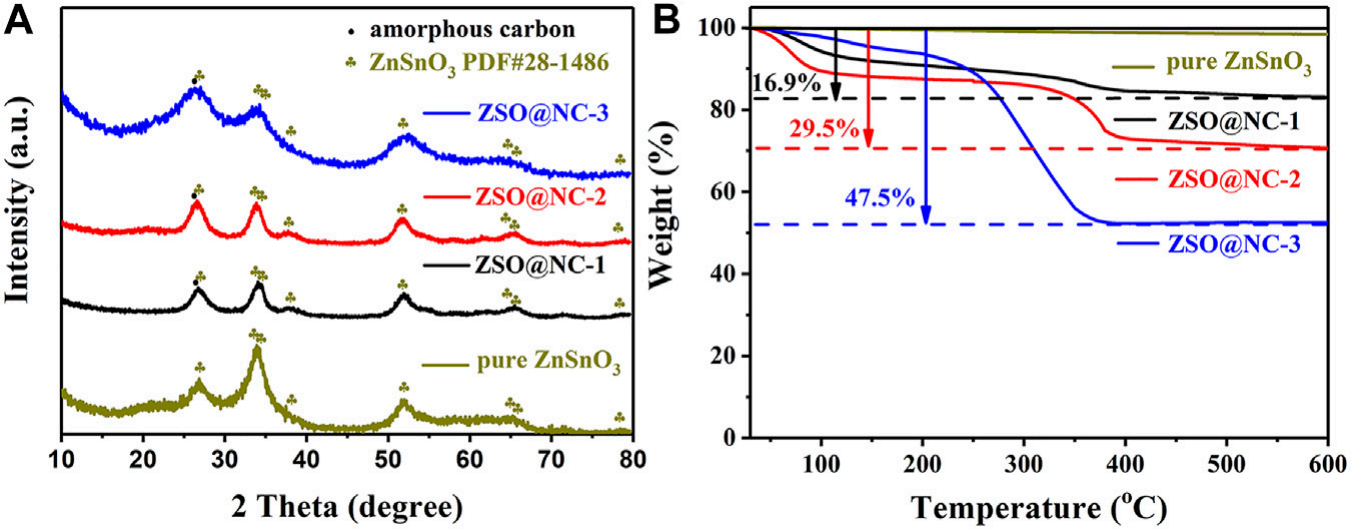
XRD patterns **(A)** and TGA curves **(B)** of the ZSO@NC-(1,2,3) and pure zinc stannate (ZnSnO_3)._

Figure 1 illustrates the synthesis procedure of ZSO@NC nanocomposites with the core–shell structure. First, ZnSn(OH)_6_ precursors were obtained by co-precipitation method under ice-bath conditions. Then, PPy was coated on the surface of ZnSnO_3_ nanoparticles by *in situ* polymerization under ice-bath conditions. Finally, the ZSO@NC composites were obtained by calcination in an inert atmosphere. Through optimizing the synthesis conditions, the composites with special core–shell structure were obtained. The XRD patterns of ZnSn(OH)_6_ are shown in Supplementary Figure S1, with all the diffraction peaks corresponding to the standard peaks of ZnSn(OH)_6_ (JCPDS-No. 20-1455). Figure 2A displays the XRD patterns of ZSO@NC-(1,2,3) and pure ZnSnO_3_. These diffraction peaks can basically be indexed to the standard cards ZnSnO_3_ phase (JCPDS-No. 28-1486), indicating partial ZnSn(OH)_6_ had transformed to the ZnSnO_3_ phase under calcination conditions (Ma et al., 2018). In addition, with the increase in pyrrole content in the above four samples, the carbon peak at 26.5° was significantly enhanced, indicating the presence of amorphous carbon (Liu et al., 2020; He et al., 2017). Figure 2B shows the thermogravimetric analysis (TGA) of ZSO@NC-(1,2,3) and pure ZnSnO_3_. It revealed that the amount of NC in the ZSO@NC-(1,2,3) composites is about 16.9, 29.5, and 47.5 wt%, respectively, and there is little mass loss in the pure ZnSnO_3_. Supplementary Figure S2 shows the Raman spectra of all the composites. The difference between ZSO@NC-(1,2,3) and pure ZnSnO_3_ is that there are two main peaks located at ∼1,350 and ∼1,557 cm^−1^, which can be attributed to the D peak of amorphous carbon and the G peak of graphitized carbon. For NC@ZSO-2, the intensity ratio of D peak to G peak is 1.02. This indicates that there are lots of vacancies and graphitized carbon between the layers of carbon in this material (Yu et al., 2018). The surface elemental compositions and electronic states of ZSO@NC-2 were characterized by XPS measurement. The deconvolutions of the C1s spectra are exhibited in Supplementary Figure S3A. The peaks at 284.3, 285.4, and 287.6 are associated with C-C, C=C, and C-N functional groups, respectively. In Supplementary Figure S3B, the N1s spectra can be deconvoluted into two individual peaks that are assigned to pyridinic N (398.0 eV) and pyrrolic N (399.8 eV) (Hu et al., 2013). Supplementary Figure S3C shows the high-resolution XPS spectrum of the Zn 2p. The two photoelectron peaks at binding energies of 1,021.8 and 1,044.9 eV correspond to Zn 2p3/2 and Zn 2p1/2, respectively. For the spectrum of Sn 3d, the two peaks observed at 486.9 and 495.4 are related to Sn 3d5/2 and Sn 3d3/2, respectively, as observed in Supplementary Figure S3D. It manifests the existence of Zn^2+^ and Sn^4+^ in the ZSO@NC-2 particles (Liang et al., 2018). The XPS results further verify the coexistence of ZnSnO_3_ and NC in the composite, which agrees well with the XRD results.

**FIGURE 3:**
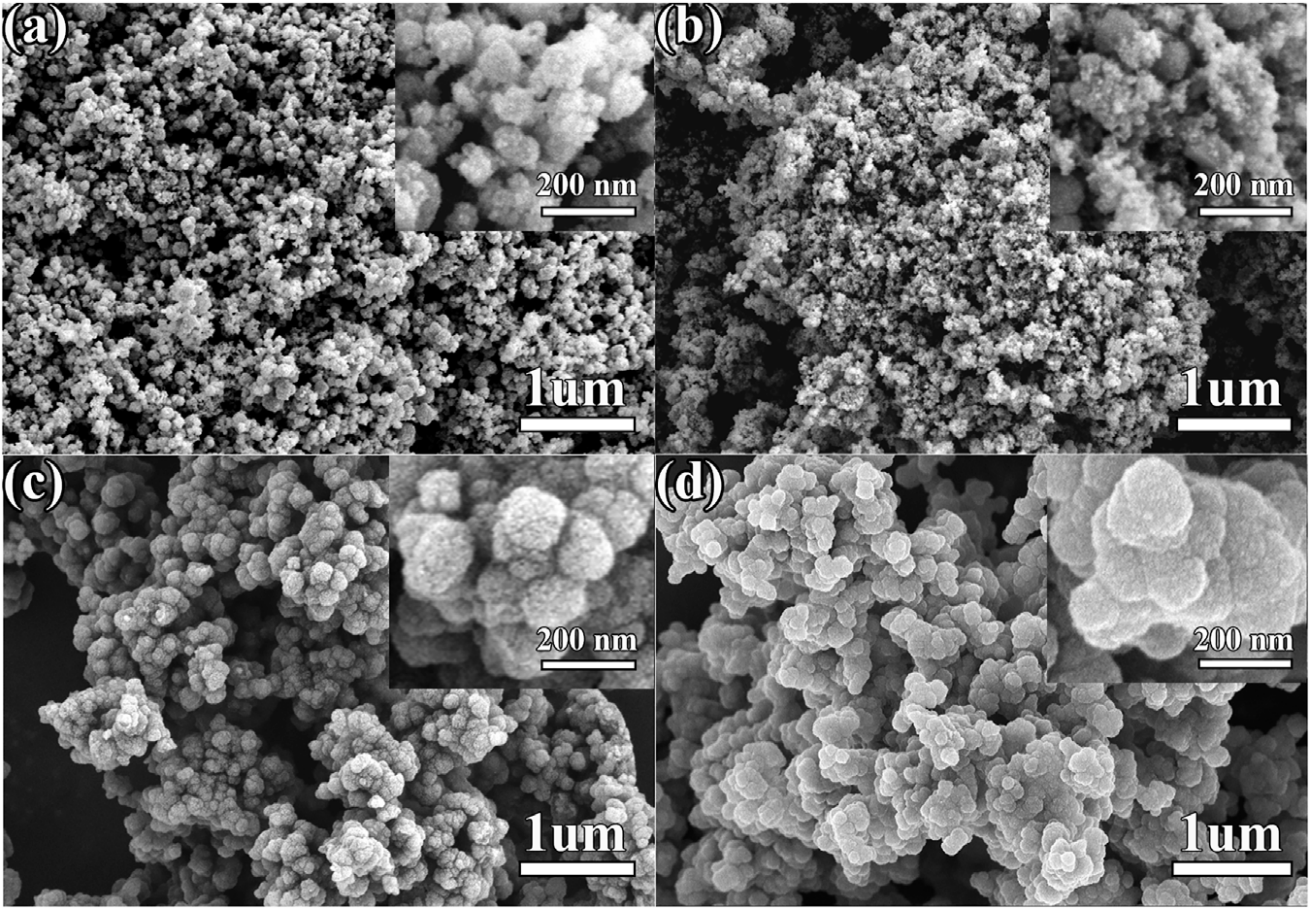
FE-SEM image of pure ZnSnO_3_ **(A)**, ZSO@NC-1 **(B)**, ZSO@NC-2 **(C)**, and ZSO@NC-3 **(D)**.

**FIGURE 4:**
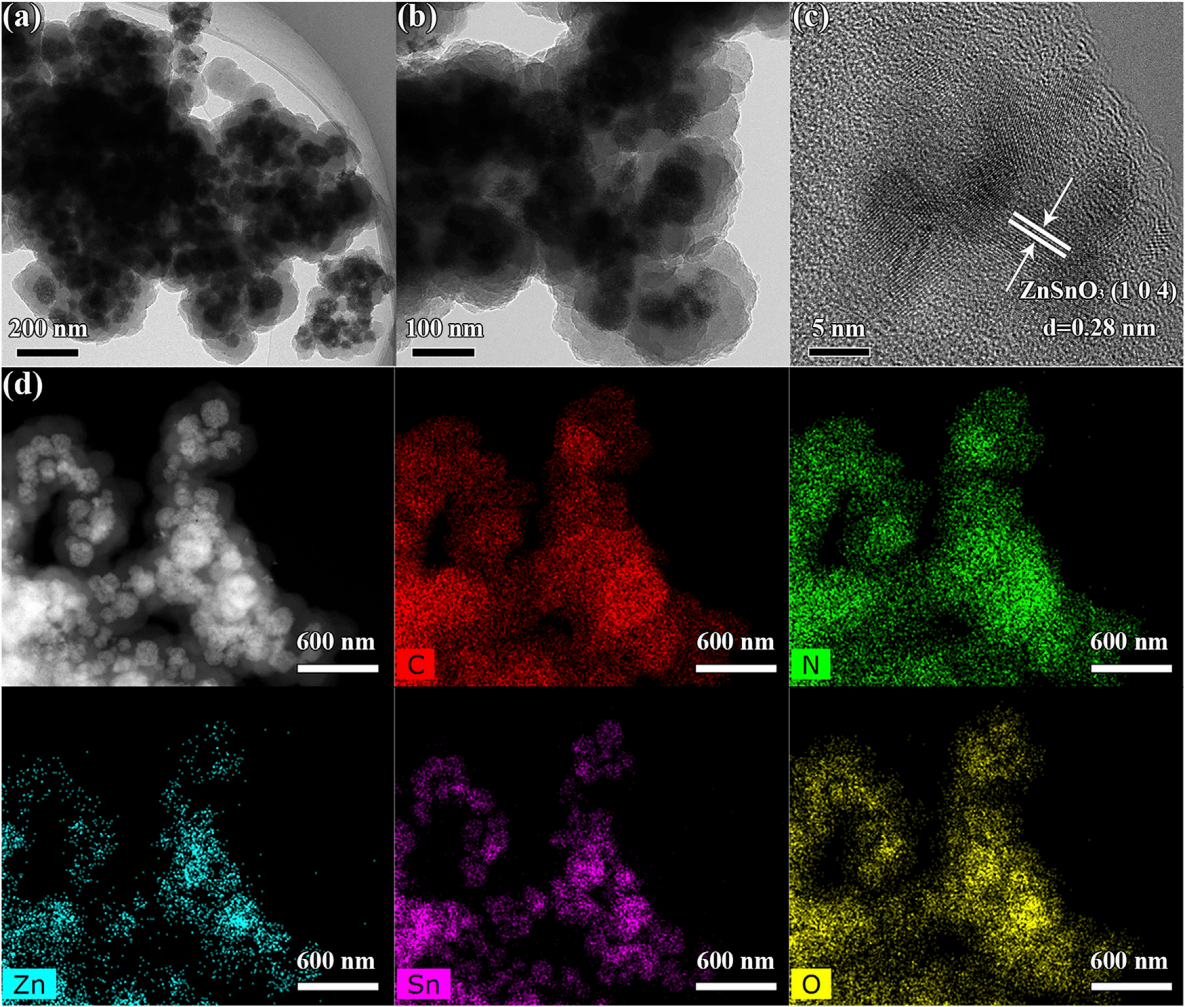
TEM **(A, B)** and HRTEM **(C)** and EDS mapping **(D)** images of NC@ZSO-2.

Special FE-SEM images provide insight into the morphologies and structures of the as-synthesized ZSO@NC-(1,2,3) and pure ZnSnO_3_. As shown in Figure 3A, pure ZnSnO_3_ are numerous homogeneous nanoparticles, and the average size of nanoparticles is estimated from a statistic of 200 samples to be about 80.5 nm (Supplementary Figure S4A). The FE-SEM images of ZSO@NC-2 is observed in Figure 3B, it revealed that the surface of the ZnSnO_3_ nanoparticle is covered with many smaller particles. By comparing Figures 3A, B, it can be inferred that the smaller nanoparticle is carbon. As displayed in Figure 3C, the ZSO@NC-2 is composed of large particles, which are agglomerated one by one. After NC coating, the average size of nanoparticles is significantly increased to 181.4 nm estimated from a statistic of 200 samples (Supplementary Figure S4B). Compared with Figure 3C, the particles in Figure 3D are larger, and the agglomeration phenomenon is more serious, which may be caused by the thicker carbon layer formed by a higher carbon content in ZSO@NC-3. The structural and morphological characteristics of ZSO@NC-2 are further revealed by TEM, HRTEM, and EDS mapping in Figure 4. As shown in Figures 4A, B, ZSO@NC-2 has a unique core–shell structure, consisting of a carbon layer completely encapsulated with ZnSnO_3_ particles. Figure 4C displays the corresponding HRTEM image, and these nanoparticles are crystalline with the fringe spacing of 0.28 nm, corresponding to the (104) plane of ZnSnO_3_ (Zhou et al., 2017). Figure 4D is the EDS-Mapping image of ZSO@NC-2. It can be found that the distribution of C and N elements is exactly the same, indicating that this carbon layer is composed of nitrogen-doped carbon (NC). The elemental distribution of Zn, Sn, and O is basically the same. Meanwhile, the elemental distribution range of C and N completely covers that of Sn, Zn, O element, which further confirms that ZSO@NC-2 is a core–shell structure composed of NC completely coated ZnSnO_3_ particles.

**FIGURE 5:**
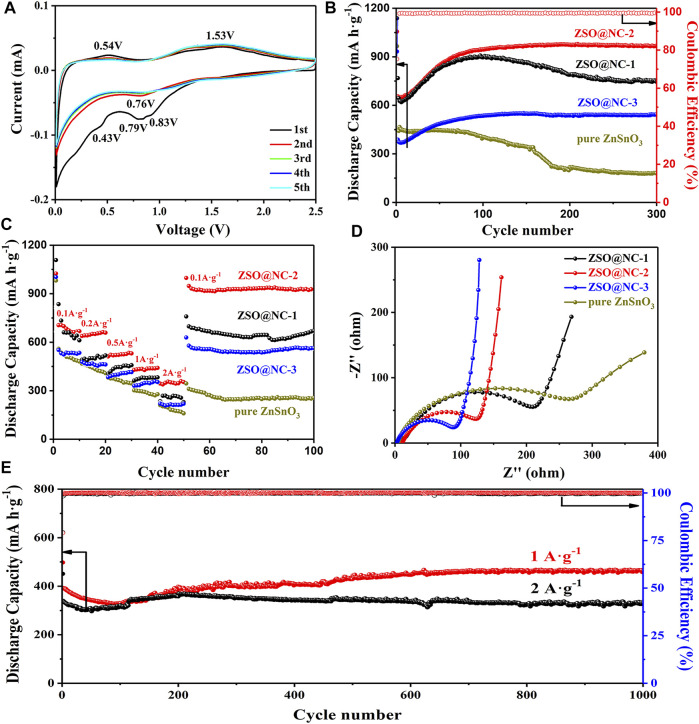
**(A)** Typical CV curves of ZSO@NC-2, **(B)** Cyclic performance curves of ZSO@NC-(1,2,3) and pure ZnSnO_3_ at 0.1 A g^−1^. **(C)** Specific capacity versus current densities. **(D)** EIS curves of ZSO@NC-(1,2,3) and pure ZnSnO_3_. **(E)** Cyclic performance curves of ZSO@NC-2 at 1 and 2 A g^−1^.

The electrochemical mechanism of NC@ZSO-2 was explored by cyclic voltammetry test in Figure 5A. In the first cycle, two wide inconspicuous cathodic peaks centered at 0.43 and 0.79 V, which could be attributed to the formation of SEI film and the decomposition of ZnSnO_3_ (Ma et al., 2018). Another two obvious oxidation peaks at 0.54 and 1.53 V were observed, which could be interpreted as the reaction of Li^+^ detachment from the metal alloy Li–Sn and Li–Zn. In the second and subsequent cycles, the peak of 0.79 V shifted to 0.83 V. The two redox couples, indexed as 0.05/0.53 and 0.83/1.53 V, appeared due to the alloying/dealloying process and the formation/decomposition of Li_2_O. After the first cycle, the subsequent CV curves are well overlapped, confirming the stable storage and release of Li^+^ in the ZSO@NC-2 material. Figure 5B shows a comparison of the cycling performance of the ZSO@NC-(1,2,3) and pure ZnSnO_3_ at 0.1 A g^−1^. The discharge capacity of ZSO@NC-2 decreased in the initial several cycles, then increased gradually with continuous cycling. Finally, a relatively stable discharge capacity of 967 mA h g^−1^ was achieved after 300 cycles with the CE approaching 100%. The gradual increase in the capacity may have mainly resulted from the formation of a gel-like polymeric layer and possibly interfacial lithium storage as well as electrochemical activation process of the composite during the repeated Li insertion/extraction (Yu et al., 2018; Hu et al., 2013; Liang et al., 2018). To highlight the advantage of bimetallic oxide, ZnO-SnO_2_@NC is obtained at 800°C for 2 h at a heating rate of 5°C/min under a protective atmosphere. Except for the different calcination conditions, the preparation process of ZnO-SnO_2_@NC is identical with that of ZSO@NC-2. Similarly, nitrogen-doped carbon (NC) is prepared by imitating the production process of ZSO@NC-2 without adding zinc source and tin source. As shown in Supplementary Figure S5A, ZnO-SnO_2_@NC is mainly composed of SnO_2_ (PDF#24–1,470), Zn_2_SnO_4_ (PDF#41–1,445), and amorphous carbon. The cyclic performance of the above materials is tested at 0.1 A·g^−1^ by assembling into the Li^+^ half-battery (Supplementary Figure S5B). Compared with ZnSnO_3_, the cycle stability of ZnO-SnO2@NC electrode is extremely poor with only 134 mA h g^−1^ after 200 cycles, which indicates that the unique bimetal structure of ZnSnO_3_ has performance advantages. The cycling performance curve of ZSO@NC-3 is similar to that of ZSO@NC-2. Due to the high NC content of the former, the specific capacity of the former is lower than that of the latter after stabilization. The reversible capacity of ZSO@NC-3 is 544 mA h g^−1^ after 300 cycles. Compared with the above two samples, ZSO@NC-1 has a lower cyclic stability, attributing to the lower NC content. The reversible capacity of ZSO@NC-1 is 745 mA h g^−1^ after 300 cycles. Compared with the previous three samples, pure ZnSnO_3_ exhibit extremely worst cycle stability (187 mA h g^−1^ after 300 cycles), which can be attributed to the violent volumetric expansion effect during the repeated Li insertion/extraction process. Nitrogen-doped carbon (NC) is successfully prepared by imitating the production process of ZSO@NC-2 without adding zinc source and tin source. As shown in Supplementary Figure S6A, NC is mainly composed of amorphous carbon. The NC electrode shows excellent cycling stability (Supplementary Figure S6B). However, its specific capacity is only about 250 mA h g^−1^, which is too low to compare with that of ZSO@NC-2, and NC (in Supplementary Figure S6D) also shows a good rate capability. The corresponding reversible capacities were 301, 252, 205, 177, and 152 mA h g^−1^ at 0.1, 0.2, 0.5, 1, and 2 A g^−1^ after 10 cycles, respectively. As shown in Figure 5C, ZSO@NC-2 showed the best rate capability among the as-synthesized samples. The corresponding reversible capacities were 698, 641, 521, 436, and 362 mA h g^−1^ at 0.1, 0.2, 0.5, 1, and 2 A g^−1^ after 10 cycles, respectively. In contrast, pure ZnSnO_3_ only delivered 480, 409, 344, 278, and 169 mA h g^−1^ at the same current densities, respectively. When the current density recovered from 2 to 0.1 A g^−1^, ZSO@NC-2 delivered an even higher capacity than the primary capacity at various current densities, indicating good reproducibility. The excellent cyclic performance of ZSO@NC-2 can be attributed to its unique compact core–shell structure. The shell formed by nitrogen doping carbon cannot only effectively alleviate the volume expansion effect of ZnSnO_3_ but also enhance the conductive property of the material, thus, greatly improving the lithium storage performance of the material. Figure 5D reveals the Nyquist plots of ZSO@NC-(1,2,3) and pure ZnSnO_3_. In general, the electrolyte resistance (R_s_) corresponds to the first intercept of semicircle on the Z′ axis, the charge transfer resistance (R_ct_) is determined by the diameter of the semicircle, and the resistance of Li ion diffusion (Z_w_) is related to the linear slope (Yu et al., 2018). The R_s_ and R_ct_ values of ZSO@NC-2 are 11.1 and 143.4 Ω, respectively. Oi contrast, that of pure ZnSnO_3_ is larger (11.7 and 302.1 Ω) (Supplementary Table S1). Supplementary Figure S6C reveals the Nyquist plot of NC. The R_s_ and R_ct_ values of NC are 3.5 and 13.9 Ω, respectively, indicating that the material has excellent electrical conductivity. The shell formed by nitrogen doping carbon cannot only effectively alleviate the volume expansion effect of ZnSnO3 but also enhance the conductive property of the material, thus, greatly improving the lithium storage performance of the material. The low Rct value enables faster charge transfer, achieving better rate capability of the ZSO@NC-2 electrode. To examine the longer cycling stability of ZSO@NC-2, it was further cycled to 1,000 cycles at a higher current density of 1 and 2 A g^−1^. As shown in Figure 5E, when cycled at 1 and 2 A g^−1^, the reversible capacity remained stabilized at 458 and 365 mA h g^−1^ after 1,000 cycles. The cyclic failure mechanism of materials with high specific capacity can be attributed to the severe volume expansion effect generated during the cyclic process, which will continuously destroy SEI, consume electrolytes to form new SEI, and eventually lead to extremely poor cyclic stability. As shown in Supplementary Figure S6, the NC electrode shows good conductivity and excellent cycle stability, which indicates that the NC electrode can easily form a stable SEI layer. A large number of studies have shown that the core–shell structure can effectively improve the cyclic stability, with NC as the shell and high specific capacity active substance as the core. On the one hand, the NC shell can alleviate the volume expansion effect. On the other hand, the NC layer can effectively separate the active substance from the electrolyte and contribute to the formation of the stable SEI layer. What is more, the excellent electrical conductivity of the NC shell also contributes to the formation of the stable SEI layer, which greatly improves cycle stability.

## Conclusion

In this paper, a core–shell ZSO@NC nanocomposite was successfully obtained by coating ZnSnO_3_ with polypyrrole (PPy) through *in situ* polymerization under ice-bath conditions. Through a series of physical characterization, it revealed that the composite has a unique core–shell structure. The shell is composed of nitrogen-doped carbon spheres, and the core is composed of cubic ZnSnO_3._ Benefiting from this unique compact structure, ZSO@NC-2 delivers an excellent lithium storage performance. When the current density is 0.1 A g^−1^, the ZSO@NC can deliver a reversible capacity of 967 mA h g^−1^ after 300 cycles, which is nearly five times that of pure ZnSnO_3_. When the current density increases to 1 and 2 A g^−1^, the reversible capacity of ZSO@NC-2 can reach 458 and 365 mA h g^−1^ after 1,000 cycles, respectively.

## Data Availability

The original contributions presented in the study are included in the article/Supplementary Material. Further inquiries can be directed to the corresponding authors.
