# Supplementary material for: Nitrogen-Doped Carbon Encapsulated Partial Zinc Stannate Nanocomposite for High-Performance Energy Storage Materials
Source: Front Chem. 2021 Nov 18;9:769186. doi: 10.3389/fchem.2021.769186 (PMC8636980; doi:10.3389/fchem.2021.769186)
Supplement: Supplementary file 1 [file DataSheet1.pdf]

SUPPLEMENTARY MATERIAL

Preparation of Core-shell $ZnSnO_3$@NC nanocomposites for Advance Li-ion Battery anode materials

Jiage Yu, Yu Ding, Xian Zhang, Zhijie Liu, Feng Wang*

School of Chemistry and Materials Science, Hubei Engineering University, Xiaogan 432000, Peoples Republic of China.

* *Corresponding authors. Email: yujiage5@163.com. (J.G Yu), hbeukj@126.com(F, Wang).*

1 Battery fabrication

The electrochemical performances of above four samples were evaluated in the CR 2016-coin cells. The test electrode was fabricated by mixing the active material, acetylene black (Super P) and poly-vinylidene fluoride (PVDF) binder in a weight ratio of 80: 10: 10 in N-methyl-2-pyrrolidine (NMP) solution. Then, the above slurry was spread evenly over the copper foil and dried at 80 ºC in vacuum oven for 12 h. The average mass of the active material in the electrode is ~1.5 mg cm^{-2}. These cells were assembled by using an electrolyte solution of 1M $LiPF_6$ in dimethyl carbonate (DMC) and ethylene carbonate (EC) (1:1 by volume) as the electrolyte, lithium foil as the counter electrode and Cellgard membrane as the separator. Before test, these cells were placed at 25 ºC for 12 h to ensure the complete infiltration of the electrolyte.

2 Characterization

The X-ray diffraction (XRD) analysis was performed by using an X'Pert Pro diffractometer with Cu K_α radiation (λ=0.154 nm). The Raman spectra was observed by a DXR Raman microscope (Thermo Fisher Scientific), using He-Ne 532 nm laser excitation. The thermogravimetric analyzer (Netzsch STA449 F5) was used to measure the carbon content in the composite materials. The microstructure and morphology were observed by transmission electron microscopy (TEM, FEI, Tecnai G20) and FE-SEM (JEOL, 6510V). Elemental analysis was conducted on the energy dispersive X-ray spectroscopy (EDS, Oxford X-Max 80 SSD) detector attached to TEM. Cyclic voltammetry (CV) profiles were obtained by a CHI660E electrochemical workstation. Galvanostatic charging/discharging test was conducted in the potential window of 0.01-2.5 V on Landt CT2001A battery test systems.

3. Figure

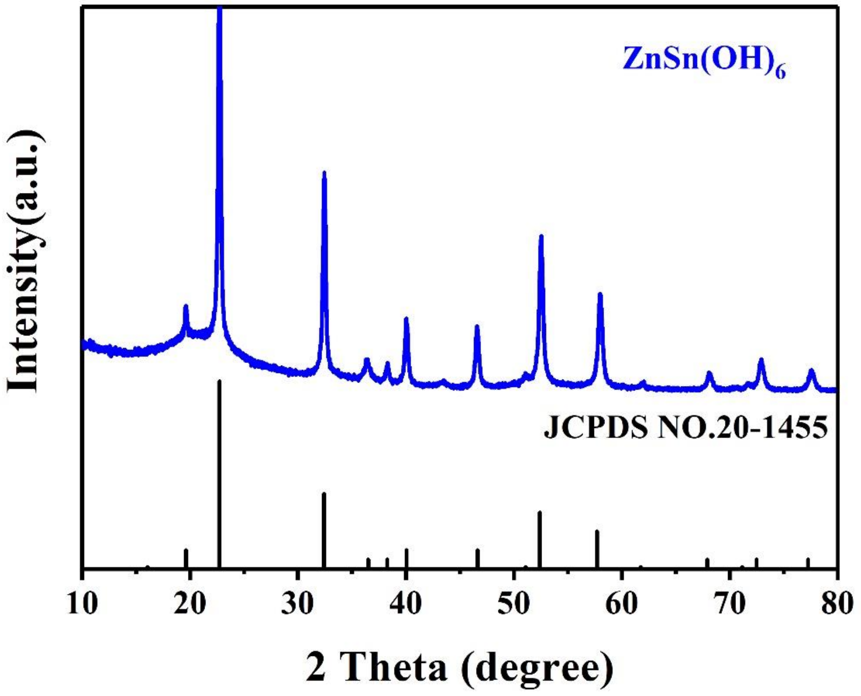

Fig. S1 XRD patterns of $ZnSn(OH)_6$.

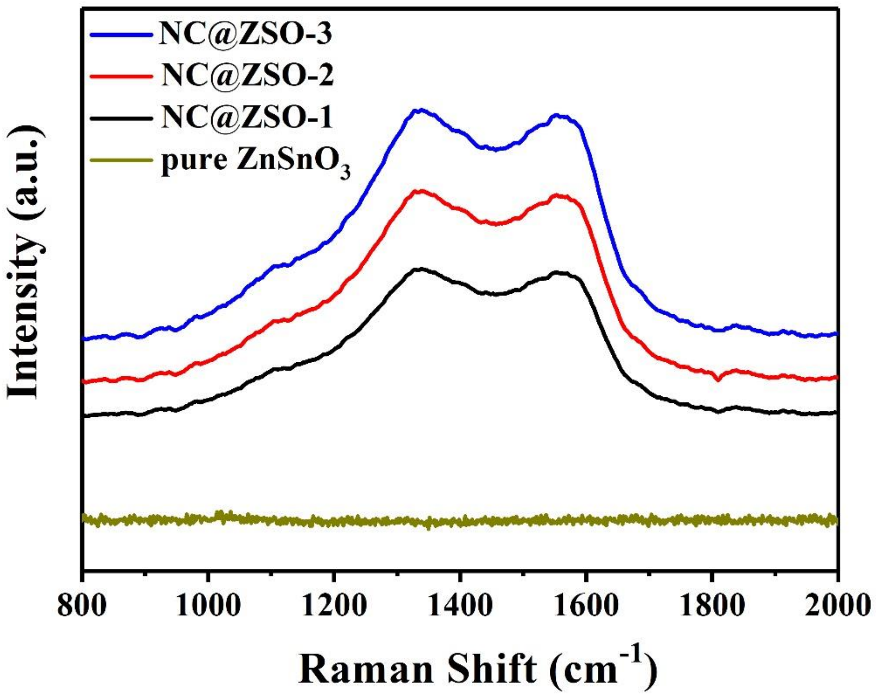

Fig. S2 Raman spectra (b) of the ZSO@NC-(1,2,3) and pure $ZnSnO_3$.

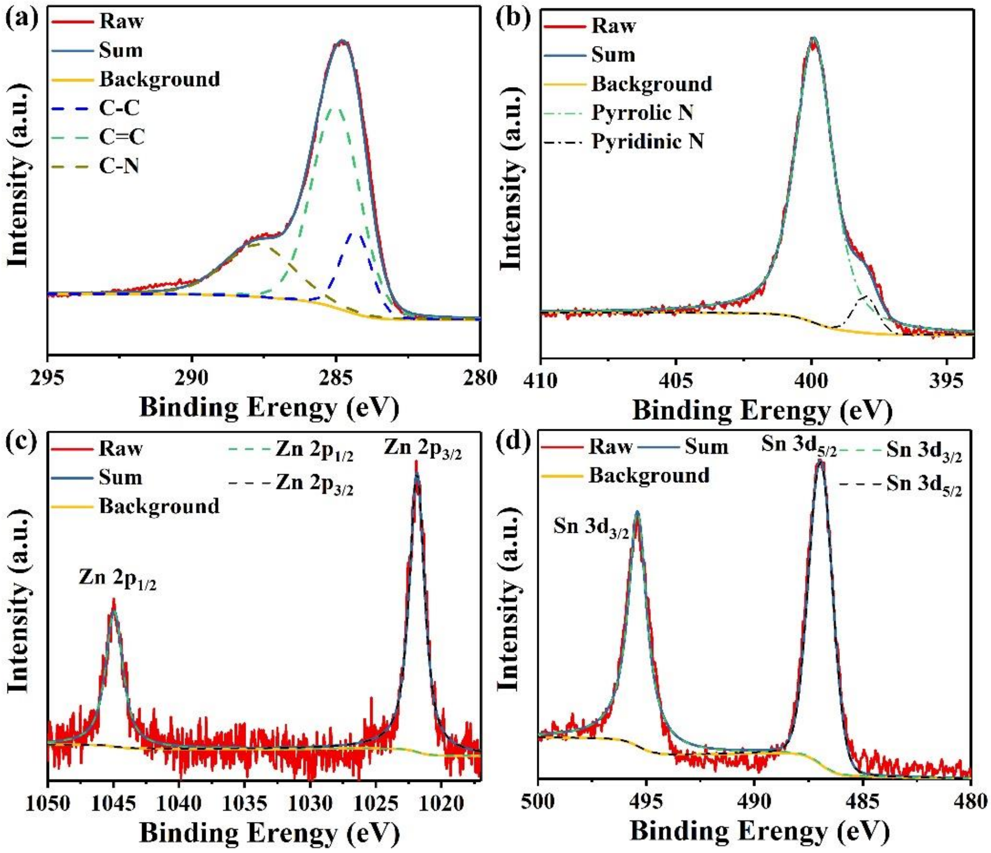

Fig. S3 (a) C 1s, (b) N 1s (c) Zn 2p and (d) Sn 3d high-resolution XPS spectra of NC.

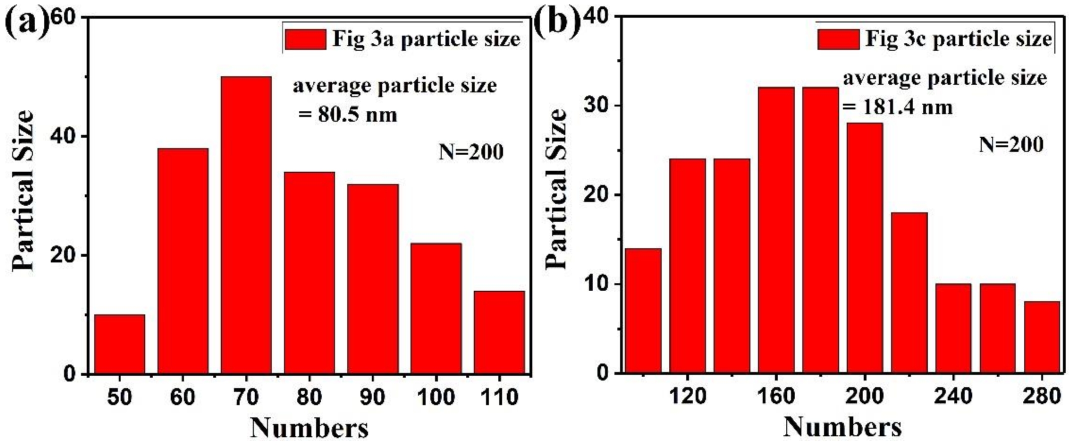

Fig. S4 Particle size distribution of figure 3a (a), figure 3c (b).

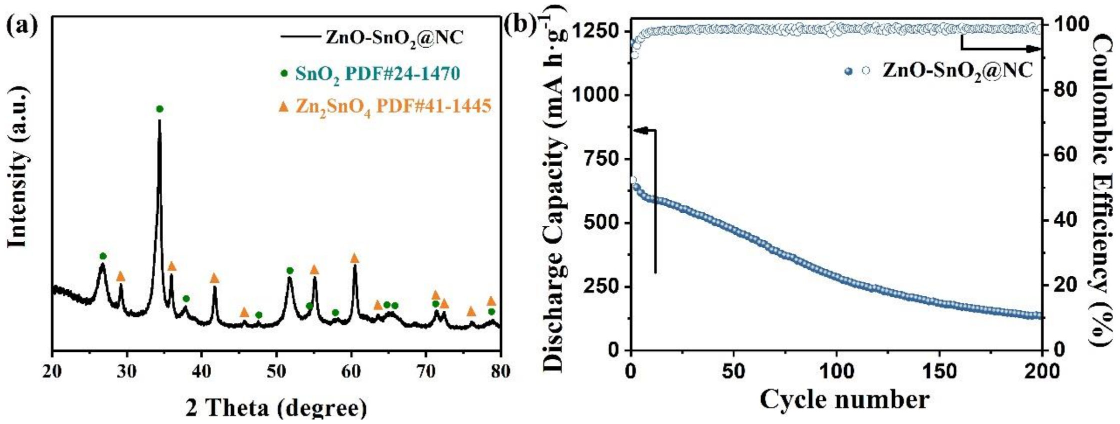

Fig. S5 (a) XRD patterns and (b) Cyclic performance curves at 0.1 A·g^{-1} of ZnO-SnO_2@NC.

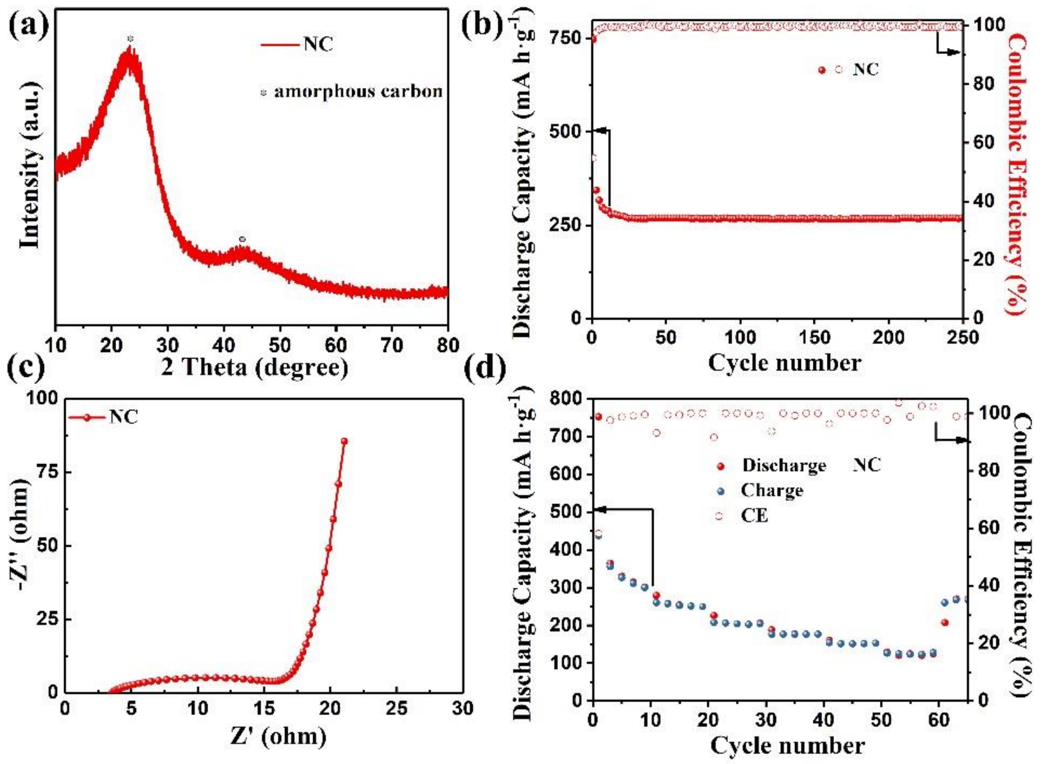

Fig. S6 (a) XRD patterns, (b) Cyclic performance curves at 0.1 A·g^{-1}, (c) EIS curve and (d) Specific capacity versus current densities of NC.

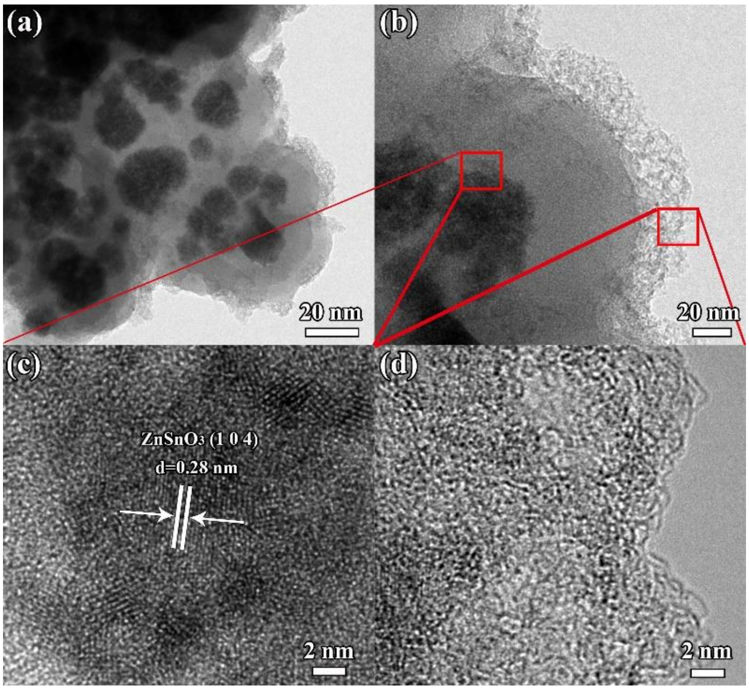

Fig. S7 (a, b) TEM and (c, d) HRTEM images of NC@ZSO-2 after 300 cycles at 0.1 $A \cdot g^{-1}$.

4. Table

Table S1. EIS parameters obtained from EIS map fitting of the sample

sample	$R_s(\Omega)$	R_{ct} (Ω)
ZSO@NC-1	3.2	79.6
ZSO@NC-2	11.1	143.4
ZSO@NC-3	3.3	219.4
pure $ZnSnO_3$	11.7	302.1
NC	3.5	13.9
